# Regulatory T cells are less sensitive to glucocorticoid hormone induced apoptosis than CD4^+^ T cells

**DOI:** 10.1007/s10495-020-01629-x

**Published:** 2020-07-31

**Authors:** Lilla Prenek, Tímea Litvai, Noémi Balázs, Réka Kugyelka, Ferenc Boldizsár, József Najbauer, Péter Németh, Timea Berki

**Affiliations:** Department of Immunology and Biotechnology, Clinical Center, Medical School, University of Pécs, Szigeti út 12, Pécs, 7624 Hungary

**Keywords:** Glucocorticoid hormone, Dexamethasone, Thymocytes, Splenocytes, CD4^+^ Th cells, pTreg, tTreg, Apoptosis, Caspase, Ca^2+^ signaling

## Abstract

Earlier we have reported that thymic regulatory T cells (Treg) are resistant to in vivo glucocorticoid hormone (GC)-induced apoptosis, while the most GC-sensitive DP thymocytes died through the activation of mitochondrial apoptotic pathway. Here we analyzed the apoptosis-inducing effect of high dose (10^–6^ M) in vitro dexamethasone (DX) treatment in mouse thymic- and splenic Tregs and CD4^+^ T cells. Activation of both extrinsic and intrinsic apoptotic pathways started after 2 h of DX treatment in CD4 SP thymocytes and was 3 × higher than in CD4^+^ splenocytes, while in Treg cells, weak activation of the extrinsic apoptotic pathway started only after 3 h. We also investigated the expression of 21 apoptosis-related molecules using a protein array and found higher level of both pro-and anti-apoptotic molecules in Tregs compared to CD4^+^ T cells. 4 h in vitro DX treatment induced upregulation of most apoptosis-related molecules both in Tregs and CD4^+^ T cells, except for the decrease of Bcl-2 expression in CD4^+^ T cells. We found high basal cytosolic Ca^2+^ levels in untreated Treg cells, which further increased after DX treatment, while the specific TCR-induced Ca^2+^ signal was lower in Tregs than in CD4^+^ T cells. Our results suggest that in the background of the relative apoptosis resistance of Treg cells to GCs might be their high basal cytosolic Ca^2+^ level and upregulated Bcl-2 expression. In contrast, downregulation of Bcl-2 expression in CD4^+^ T cells can explain their higher, DX-induced apoptosis sensitivity.

## Introduction

Glucocorticoid hormones (GCs) regulate essential physiologic functions, control metabolism, growth, differentiation, and apoptosis of numerous cell types [1–3]. GCs also influence the immune functions by suppressing inflammation and cytokine production. They have multiple immune-modulatory effects involving leukocyte apoptosis and differentiation. Despite their numerous side-effects, synthetic GCs are the most prescribed drugs used for the treatment of autoimmune and inflammatory diseases, allergies, and certain types of hematological malignancies [4]. GC analogues have been shown to influence immune functions by promoting the apoptosis of immature CD4^+^CD8^+^ double positive (DP) thymocytes [5] and to trigger complex anti-inflammatory actions by influencing both the molecular and cellular components of the immune system [6]. They mediate their biological effects by binding to intracellular glucocorticoid receptors (GRs) that can act through genomic and non-genomic mechanisms. In the genomic pathway ligand-bound GR can serve as a transcription factor. It can induce or repress the transcription of numerous genes by directly binding to GC response elements (GRE) or by physically associating with other transcription factors [2]. We reported in a previous study that GC hormone treatment resulted in mitochondrial translocation of ligand-bound GR in DP thymocytes [7]. Short-term in vitro treatment with GC analogue induced interactions between the ligand-bound GR and Bcl-2 family member proteins, e.g. Bak, Bim, and Bcl-xL [8]. We also characterized the activation of different caspases as markers of apoptosis in DP thymocytes and provided evidence for the mitochondrial apoptotic pathway activation as a non-genomic mechanism in DP thymocytes [7, 8].

Regulatory T (Treg) cells are a subpopulation of T cells that play an instrumental role in maintaining tolerance to self-antigens [9–12] and in suppression of excessive immune responses after antigenic stimulation. Thereby Treg cells help in maintaining an immune homeostasis and in lowering the risk for developing autoimmune diseases and allergies [13–15]. Clinically important issues are the participation of Treg cells in the prevention of organ rejection in patients after transplantation [16], in mother’s tolerance to fetus [17], and therapeutic application in autoimmune diseases [18, 19]. In addition, Treg cells can suppress anticancer immunity, thereby hindering protective immunosurveillance of neoplasia and hampering effective antitumor immune responses in tumor-bearing hosts, thus promoting tumor development and progression [20]. Identification of the factors that are specifically expressed in Treg cells and/or that influence Treg cell homeostasis and function (e.g. glucocorticoid hormones) is important for understanding the pathogenesis of cancer and autoimmune diseases, and finding new therapeutic targets. The best-characterized subsets of Treg cells are (a) the thymus-derived natural Treg cells (tTreg) and (b) peripheral Treg cells (pTreg), also named induced Treg (iTreg). The natural tTreg cells develop in the thymus during negative selection process and primarily recognize self-antigens. Induced pTreg cells are generated from naïve CD4^+^ T cells in the periphery [11, 21]. The immunosuppressive and regulatory function of Treg cells is mediated by direct cell–cell interaction or by secretion of immunosuppressive cytokines, e.g. TGFβ [21], IL-10, or IL-35 [22]. Numerous cell types are targets of Treg cells, including CD4^+^, CD8^+^ T cells, dendritic cells, B cells, macrophages, and NK cells [23].

In a previous study we demonstrated that thymic tTreg cells are resistant to repeated high dose in vivo administration of GC hormone analogue dexamethasone (DX), in a mouse model [24]. We also showed that both thymic and splenic Treg cells produced enhanced levels of immunosuppressive cytokines IL-10 and TGFβ after in vivo DX treatment, which was accompanied by elevated Foxp3 mRNA expression that may reflect a stronger Treg lineage commitment after DX treatment. More recently, we reported on the influence of GC hormone on the in vitro differentiation of Treg cells from thymic and splenic CD4^+^ T cells under different stimulation conditions to establish methods for generating stable and functionally suppressive iTregs for future use in adoptive transfer experiments [25]. From these results, and those of other laboratories, a complex picture is emerging on the effect of GCs on the immune system [4], including the context-dependent effect of GCs on proliferation, differentiation, and apoptosis of various T cell subtypes, including Treg cells [26].

In our current study, we asked the following questions: (a) Does the GC-induced apoptosis sensitivity of thymic and splenic Treg cells differ from that of CD4^+^ thymocytes and splenocytes? (b) Which cell death pathways (extrinsic or intrinsic) are involved in mediating the apoptotic process? (c) What is the effect of GC treatment on the expression of pro- and anti-apoptotic molecules in Treg and CD4^+^ T cells? (d) How does DX influence the basal cytosolic Ca^2+^ level and the kinetics of TCR-induced Ca^2+^ signaling in thymic and splenic CD4^+^ T cells and Treg cells?

## Materials and methods

### Animals

4–6 weeks old BALB/c mice (The Jackson Laboratory, Bar Harbor, ME, USA) were used in all experiments. Animals were kept under conventional conditions and were provided with water and pelleted rodent chow ad libitum. Animal experiments were carried out in accordance with the regulations of the Animal Welfare Committee of University of Pécs (#BA 02/2000-16/2015).

### In vitro GC-analogue treatment of isolated cells

Animals were euthanized, thymi and spleens were collected. The organs were mechanically homogenized in RPMI-1640 medium (Sigma-Aldrich, Budapest, Hungary), then filtered through a nylon mesh. Cell viability was determined by trypan-blue dye exclusion test using a hemocytometer. 10^7^ cells were treated with 10^–6^ M DX (dissolved in dimethyl sulfoxide, DMSO) (both from Sigma Aldrich) in serum-free RPMI for different time intervals for the different experiments. Control samples were kept under the same conditions, for the same time, in the presence of the solvent, DMSO. The in vitro DX treatment was followed by further preparation of the cells for flow cytometry and apoptosis assay.

### Antibodies

The following antibodies were used for flow cytometry: anti-CD4-Phycoerythrin (PE) (clone# RM4-5), anti-CD8-Phycoerythrin-Cyanine5 (PE-Cy5) (clone# 53-6.7) and anti-CD25-Allophycocianine (APC) (clone# PC61) (all from BD Pharmingen, San Jose, CA, USA). For analysis of activated (cleaved) caspases rabbit anti-caspase-3 (clone# 5A1E), rabbit anti-caspase-8 (clone# D5B2) and rabbit anti-caspase-9 (all from Cell Signaling Technology, Danvers, MA, USA) were used with anti-rabbit IgG-Alexa 488 (A488) (Abcam, Cambridge, MA, USA) as secondary antibody. For sorting the cells, the following antibodies were used: anti-CD4-PE, anti-CD8-PE-Cy5, anti-CD25-APC (BD Pharmingen, San Jose, CA, USA).

### Flow cytometry

For Annexin V labeling, the cells were treated with DX for 2 and 4 h, followed by cell surface labeling with anti-CD4-PE, Anti-CD8-PE-Cy5 and anti-CD25-APC antibodies in binding buffer (PBS containing 0.1% BSA and 0.1% NaN_3_) for 30 min at room temperature (RT). After a washing step in PBS, cells were washed in Annexin V binding buffer (10 mM HEPES/NaOH, pH 7.4, 140 mM NaCl and 2.5 mM CaCl_2_ (Sigma-Aldrich)), and then incubated with 5 µL Annexin V-FITC (BD Pharmingen) in 100 µL Annexin V binding buffer for 15 min in dark at RT. Then 400 µL binding buffer was added to the samples, followed by flow cytometric analysis using a FACSCalibur flow cytometer with CellQuest Pro software (Becton Dickinson, San Jose, CA, USA). The analysis was performed by using the FCS Express 6 software. Based on their cell surface CD4/CD8/CD25 expression, the following thymocyte and splenocyte cell populations were analyzed: CD4^+^CD25^−^ and CD4^+^CD25^high+^ (CD4/CD8 DP thymocytes were excluded). The Annexin V- FITC intensity of these populations were detected in FL1 channel. Fluorescent histogram plots were used to compare the ratio Annexin V positive cells in the control and DX-treated samples.

For detection of active caspases thymocytes and splenocytes were treated with DX for 0.5, 1, 2, 3, or 6 h followed by cell surface labeling with anti-CD4-PE, Anti-CD8-PE-Cy5 and anti-CD25-APC antibodies in binding buffer (PBS containing 0.1% BSA and 0.1% NaN_3_). Then the cells were fixed in 4% paraformaldehyde (Sigma Aldrich), followed by 2 washing steps in PBS, and one washing step in permeabilization buffer [PBS containing 0.1% BSA, 0.1% NaN_3_ and 0.1% saponin (Sigma-Aldrich)]. Then cells were incubated with the unlabeled primary antibodies against active anti-caspase-3, -8, -9 in separate tubes for 1 h in permeabilization buffer. After 2 washing steps, anti-rabbit IgG-A488 was used as secondary antibody in permeabilization buffer for 1 h, followed by washing with the same buffer two times, and once with PBS. Cells were measured in a FACSCalibur flow cytometer as described above, and the CD4^+^CD25^−^ and CD4^+^CD25^high+^ cell populations were gated. The fluorescence intensity of Alexa488 staining and the ratio of caspase positive cells in the gated cell populations of the control and DX-treated samples were determined using histogram overlays.

### Magnetic sorting of CD4^+^ and CD4^+^CD25^+^ cells

**Fig. 1:**
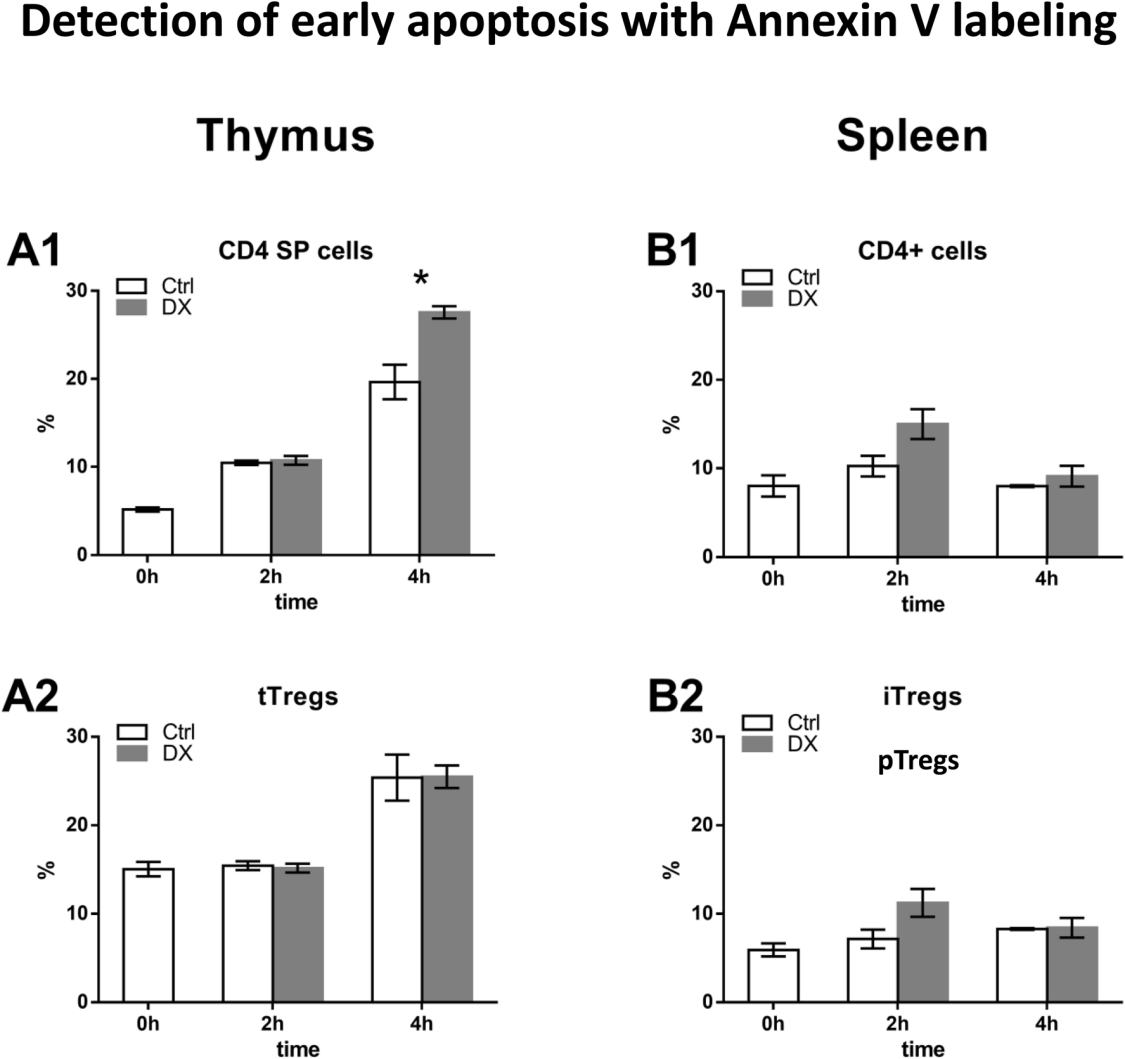
Analysis of Annexin V positivity in thymic and splenic T cell subpopulations. Cells were separated based on their cell surface CD4, CD8 and CD25 staining; Tregs were gated as CD4^+^CD8^−^CD25^high+^, CD4 SP and peripheral CD4^+^ T cells were gated as CD4^+^CD8^−^CD25^−^. Percentage of Annexin V positive cells in the gated cell populations were determined using histogram overlays. Figure shows the proportion of Annexin V positive thymic and splenic CD4^+^ T cells (**a1**, **b1**) and Tregs (**a2**, **b2**) of untreated controls (empty bars) and in 2 and 4 h DX-treated samples (grey bars). The graphs show the mean ± SEM of at least three independent experiments (n = 9). Asterisk indicates significant change (*p* < 0.05)

For sorting of each cell type (CD4 SP, CD4^+^ and pTreg), thymocytes and splenocytes from 3 mice were pooled. CD4^+^ T cells were isolated by negative selection using EasySep Mouse CD4^+^ T Cell Enrichment Kit (Stemcell Technologies, Vancouver, Canada) as previously described [24]. In some experiments the CD4^+^ T cells were subjected to further positive selection to obtain CD4^+^CD25^+^ Treg cells using EasySep™ Mouse Treg Positive Selection Kit (Stemcell Technologies, Vancouver, Canada) following the manufacturer’s instructions. The purity of the sorted Treg subpopulations was determined by intracellular staining of the cells with anti-Foxp3-PE antibody using the Foxp3 staining kit (Becton Dickinson) (Fig. 1).

### Apoptosis array

Proteome Profiler Mouse Apoptosis Array kit (R&D Systems, Minneapolis, MN, USA), a membrane-based sandwich immunoassay was used to detect the apoptosis related molecules. Capture antibodies spotted in duplicates bind specific target proteins present in the sample. Captured proteins are detected with biotinylated detection antibodies and then visualized using chemiluminescent detection reagents. The separated CD4^+^ T cells and pTreg cells were lysed in Lysis Buffer 17 (10^7^/mL cells) supplemented with 10 μg/mL Aprotinin, 10 μg/mL Leupeptin, and 10 μg/mL Pepstatin for 30 min at 4 °C. Cell lysates were centrifuged at 14,000×*g* for 5 min and the supernatant was incubated overnight at 4 °C. Then the array membranes were washed for 3 × 10 min, followed by the addition of 20 µL Detection Antibody Cocktail diluted in 200 µL distilled water and 1 mL Array Buffer for 1 h at room temperature (RT). After 3 washing steps 1:2000 diluted Streptavidin-HRP was added for 30 min at RT. After 3 washing steps, 0.5 mL of Chemiluminescent Reagent Mix was pipetted on the membrane and the reaction was detected after 5–10 min using an Image Reader LAS-4000. The dots were analyzed using the ImageJ program. The average signals from the duplicate spots were calculated and the background signal from the negative control spots were subtracted. Relative optical density was calculated by dividing the average OD of the analyte by the OD of the reference spots.

### Ca^2+^ signal measurements

For measuring intracellular Ca^2+^ signal, thymocytes and splenocytes were stained with anti-mouse-CD4-PECy5 (clone RM4-5), anti-CD8-PE (clone 53–6.7) and anti-CD25-APC (clone PC61) antibodies (all from BD Pharmingen) in dark, at RT for 30 min. After two washing steps in PBS cells (6 × 10^6^) were loaded with Ca^2+^ sensitive Fluo 3-AM dye (10 mM) (in DMSO, Sigma-Aldrich) supplemented with Pluronic F-127 (Sigma-Aldrich) for 15 min in dark at RT according to the manufacturer's instructions (Invitrogen). Cells were washed and then kept in Ca^2+^ (1.8 mM) supplemented media (RPMI containing 10% FCS) for a further 30 min to allow complete de-esterification of intracellular Fluo 3-AM esters.

For cell stimulation, purified hamster anti-mouse-CD3ε monoclonal antibody (10 μg/mL) (IgG clone 1452C11, R&D Systems), followed by goat anti-hamster-IgG pAb (28 µg/mL) (Fab 5738, Abcam) was used. For nonspecific stimulation, 1 μg of ionomycin (Sigma-Aldrich) was applied. For investigation of the short-term high dose GC effect, cells were treated with 10^–6^ M dexamethasone (DX) for 30 min before Ca^2+^ signal measurements (4 mg/mL stock in PBS).

Calcium flux kinetics were recorded using BD FACS Canto II flow cytometer (Becton Dickinson). Each tube was run for 60 s to determine baseline Ca^2+^ level, then stimulating agent was added and acquisition was continued for a total of 600 s. Compensation and analysis were carried out with FlowJo software, version 10 (FlowJo LLC, Ashland, OR, USA). Changes in cytoplasmic free Ca^2+^ levels were calculated as a relative value, by dividing the median fluorescence intensity (MFI) values at each time point with the values of Fluo 3-AM MFI before stimulation (baseline). Results are expressed as mean ± standard error of mean (SEM).

### Statistical analysis

Results were calculated from at least three independent experiments each involving three animals. Data are presented as mean ± SEM. GraphPad Prism (version 6.01, GraphPad Software, La Jolla, CA) program was used to create the artwork and perform the statistical analysis using Student’s t-test and ANOVA. *p* < 0.05 was considered statistically significant.

## Results

### Analysis of DX-induced apoptosis of thymic and splenic CD4^+^ T cells and Tregs using Annexin V labeling

In our previous work, we analyzed the effect of DX-treatment on the lymphocyte composition of Balb/c mice and we found that tTregs in the thymus were resistant to DX-induced apoptosis compared to other T cell subpopulations [24]. Especially DP thymocytes are the most sensitive cell population to both in vivo and in vitro DX-induced apoptosis compared to all other thymocyte and peripheral T cell subpopulations [7, 8].

In the current study, first we compared the effect of in vitro short-term high-dose DX treatment on thymic and splenic CD4^+^ T cells and Treg cells by investigating the phosphatidylserine externalization. Cells were treated with DX for 0, 2, 4 h, followed by surface staining with anti-CD4, anti-CD8, and anti-CD25 antibodies; then the cells were labeled with Annexin V (Fig. 1). Thus, we were able to analyze the thymic CD4 SP T cells and tTregs, and the splenic CD4^+^ T cells and pTregs separately. In the thymic CD4 SP T cells Annexin V positivity significantly increased after 4 h of DX treatment (Fig. 1a1). We have not observed the apoptosis inducing effect of DX in the case of thymic tTregs at any time point (Fig. 1a2). The Annexin V positive splenic CD4^+^ T cells and pTregs increased, but significant changes as a result of 2 or 4 h of DX treatment (Fig. 1b1, b2) could not be observed. It is interesting that in the untreated, control tTregs at 0 time point the proportion of Annexin V positive cells was higher (15%) than in the other cell populations (5–8%). At all-time points investigated, we detected higher Annexin V positivity in control, untreated tTregs and CD4 SP thymocytes compared to splenic pTreg cells and CD4^+^ T cells. This elevated apoptosis tendency could not be observed in the control splenic CD4^+^ T cells and pTegs. We can only speculate that this phenomenon could be the result of the in vivo induced apoptotic program in CD4 SP thymocytes and tTreg cells during the negative selection process in the thymus.

### Analysis of DX-induced caspase activation in thymocytes and splenocytes

Our previous results showed that short-term (30 min) in vitro DX treatment caused mitochondrial translocation of the glucocorticoid receptor (GR) in DP thymocytes. We observed direct association of ligand-bound GR with Bim and Bcl-xL resulting the intrinsic apoptotic pathway activation [8]. Here, we wanted to clarify, which apoptotic pathways are activated as a result of DX treatment in CD4 SP thymocytes and tTreg cells and compare to splenic CD4^+^ T cells and pTreg cells.

**Fig. 2:**
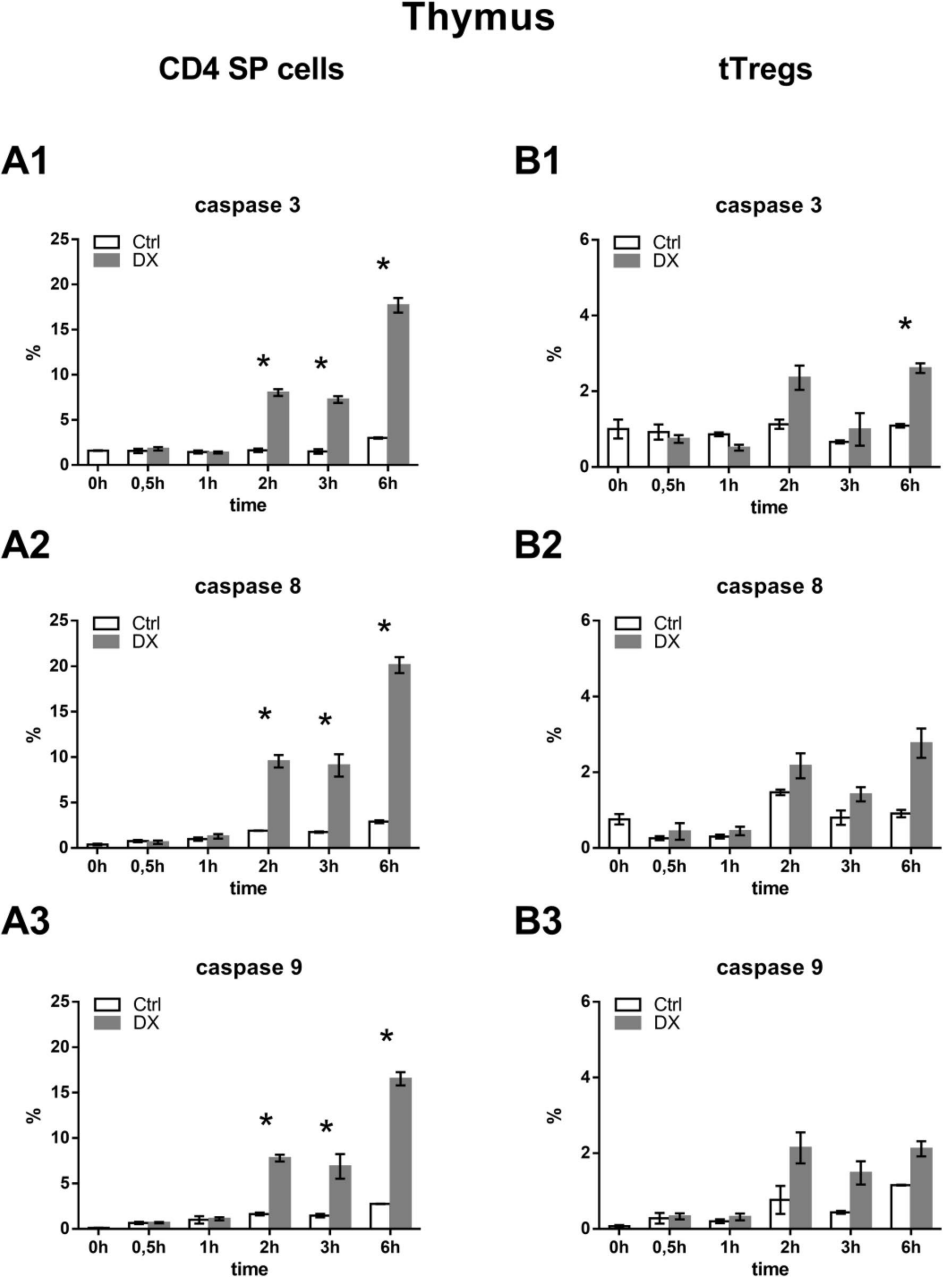
Analysis of active caspases in thymocytes with flow cytometry. CD4 SP cells and Tregs in the thymus were determined based on their CD4, CD8 and CD25 expression; Tregs were gated as CD4^+^CD8^−^CD25^high+^, CD4 SP cells were gated as CD4^+^CD8^−^CD25^−^. The proportion of active caspase containing cells in the different cell populations was defined after 0, 0.5, 1, 2, 3, and 6 h of DX treatment. Graphs show the ratio of active caspase-3, -8, -9 positive cells in thymic CD4 SP T cells (**a1**–**a3**) and Tregs (**b1**–**b3**) in control (open bars) and DX treated (grey bars) samples. Please note that the scale of Y-axes in panels **a1**–**a3** are different from the scale in panels **b1**–**b3**. The graphs show the mean ± SEM of at least three independent experiments (n = 9). Significant changes are indicated with asterisk (*p* < 0.05)

We applied CD4, CD8, and CD25 cell surface labeling to separate the cell populations and analyzed the proportion of active caspase-3, -8 and -9 positive cells after 0.5, 1, 2, 3 and 6 h of DX treatment. We found that both in thymic and splenic CD4^+^ T cells and Treg cells the DX-induced caspase activation started only after 2 h of treatment and the percentage of cells containing active caspases was lower than in the DP thymocytes reported previously (8). In thymic CD4 SP cells DX treatment caused activation of both extrinsic and intrinsic apoptotic pathways: caspase-8 and caspase-9 together with the effector caspase-3 were activated after 2 h and the ratio of active caspase-containing cells was significantly higher than in the untreated control cells (Fig. 2). After 6 h of treatment, the proportion of active caspase-3 positive cells increased from 3.0 to 17.0% (Fig. 2a1), active caspase-8 increased from 2.9% to 20.0% (Fig. 2a2), and the caspase-9 positive cells from 2.8% to 16.5% (Fig. 2a3). In thymic Tregs an increasing tendency of caspase activation could be observed, but neither caspase-8 nor caspase-9 activation showed significant changes (Fig. 2b2, b3), only the proportion of active caspase-3 positive cells increased significantly (from 1.1% to 2.6%) during the 6 h of DX treatment (Fig. 2b1), compared to untreated controls.

**Fig. 3:**
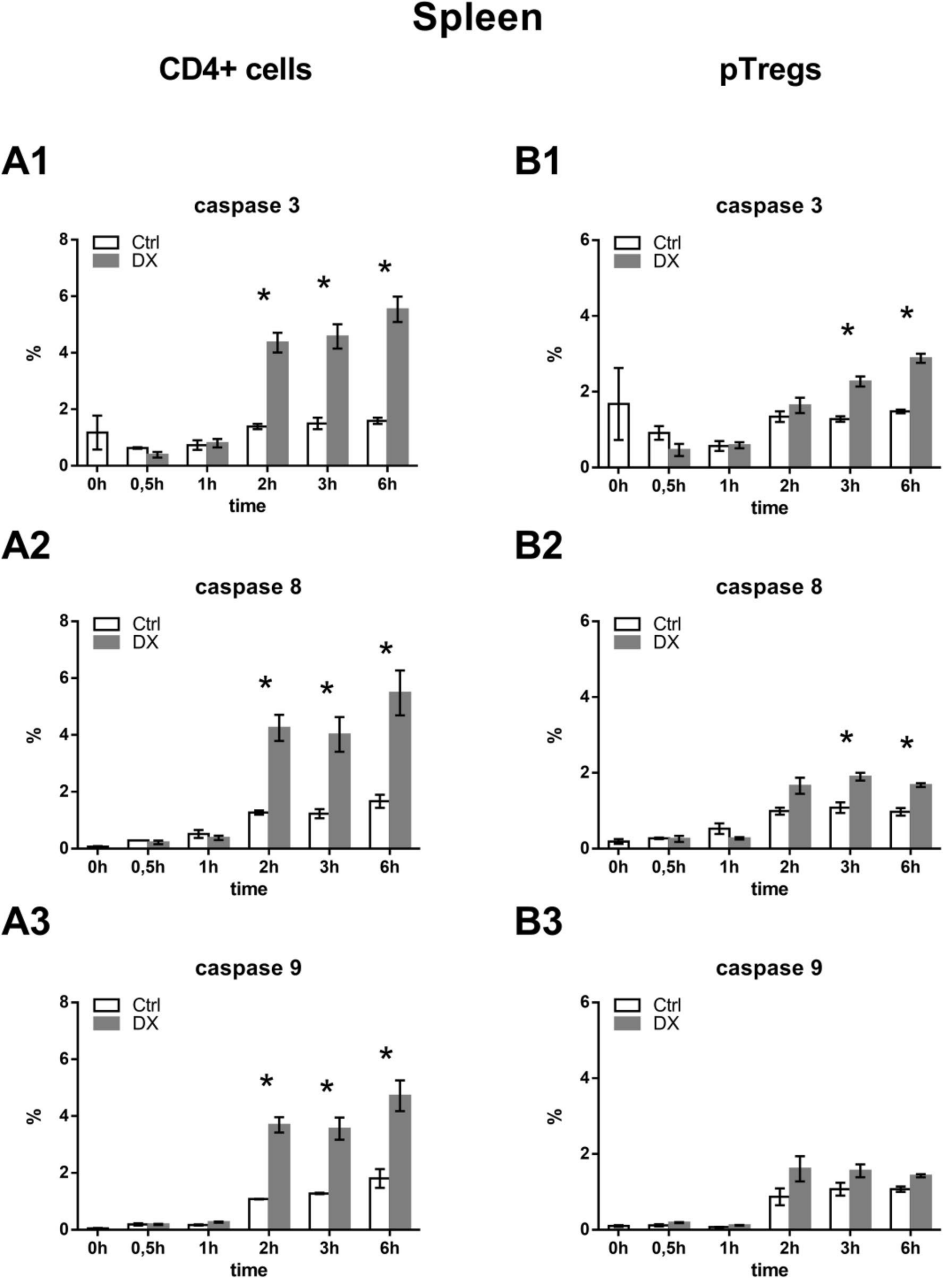
Analysis of activated caspases in splenocytes with flow cytometry. Splenic CD4^+^ T cells and pTregs were gated based on their CD4, and CD4/CD25 combined expression; Tregs were gated as CD4^+^CD8^−^CD25^high+^, CD4 SP cells were gated as CD4^+^CD8^−^CD25^−^. Percentages of active caspase containing cells in the cell populations were defined by using histograms at different time-points of DX treatment. Graphs show the percentages of active caspase-3, -8, -9 positive cells in splenic CD4^+^ T cells (**a1**–**a3**) and pTregs (**b1**–**b3**) in control (open bars) and in DX-treated samples (grey bars). The graphs show the mean ± SEM of at least three independent experiments (n = 9). Significant changes were indicated with asterisk (*p* < 0.05)

In splenic CD4^+^ T cells we found that after 2 h of DX treatment the ratio of active caspase positive cells increased significantly (Fig. 3a1–a3), and after 6 h, the proportion of caspase-3 positive cells increased from 1.6 to 5.5% (Fig. 3a1), caspase-8 from 1.7 to 5.5% (Fig. 3a2), and caspase-9 from 1.5 to 4.7% (Fig. 3a3). We would like to note that the DX-induced caspase activation in CD4^+^ splenocytes was lower at all time-points than in CD4 SP thymocytes (Figs. 2 and 3). In pTreg cells after 3 h of DX treatment we observed less pronounced, but significantly increased ratio of active caspase-3 (Fig. 3b1) and caspase-8 positive cells (Fig. 3b2), which further increased after 6 h (from 1.8 to 2.9% vs. from 1.0 to 1.7%), compared to the untreated controls. The proportion of caspase-9 positive cells did not show significant changes during the 6 h of DX treatment (Fig. 3b3). These data support that high-dose in vitro DX treatment in CD4 SP thymocytes and in CD4^+^ splenocytes resulted in both intrinsic and extrinsic apoptotic pathway activation after 2 h of treatment. Comparing the thymic and splenic Treg populations, although DX treatment caused slight activation of both extrinsic and intrinsic pathways, significant changes in the extrinsic pathway activation occurred only in pTreg cells, while in tTreg cells only the effector caspase-3 activation could be detected after 6 h of DX treatment.

### DX induced changes in the expression of apoptosis-related proteins

**Fig. 4:**
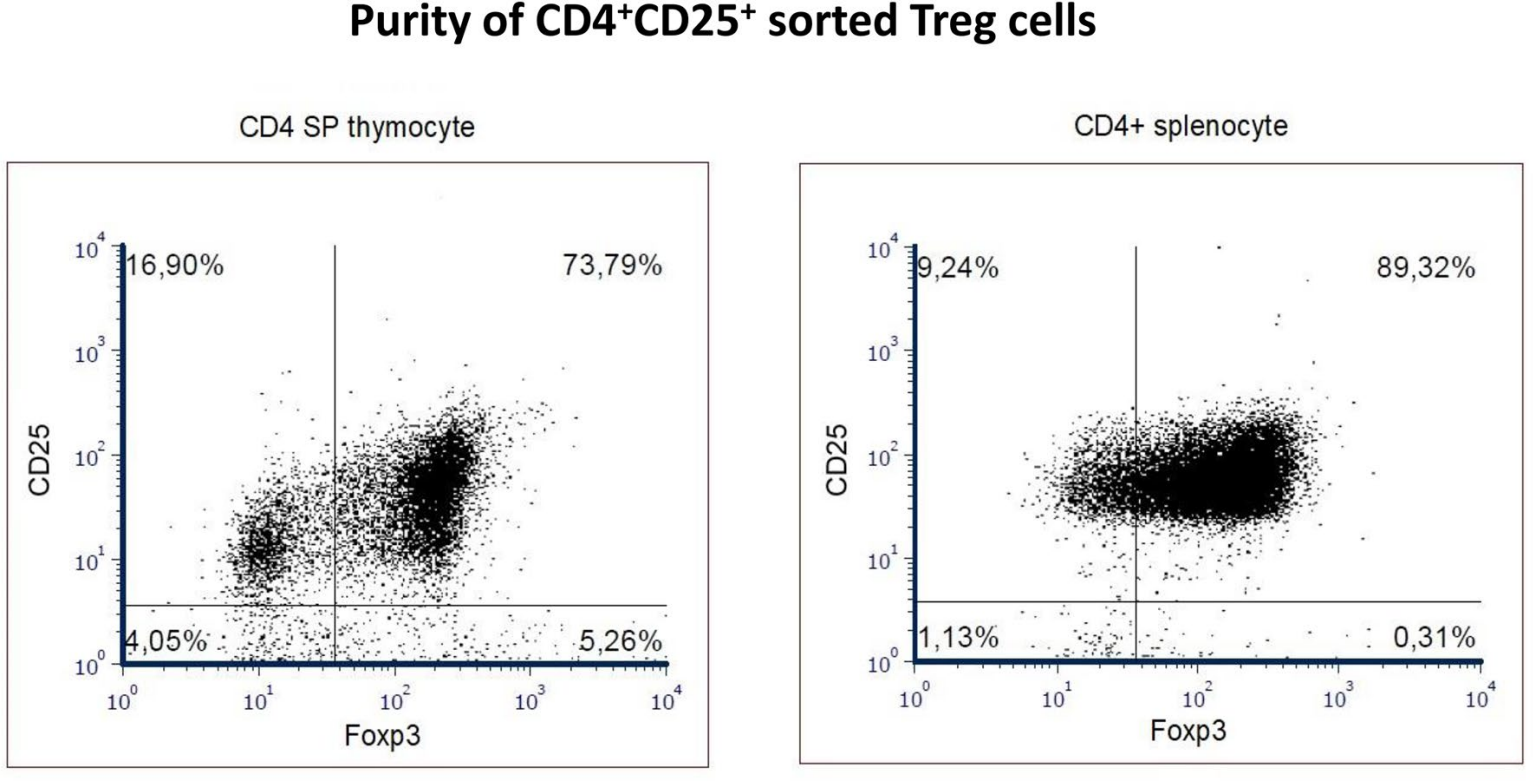
Flow cytometric analysis of the purity of sorted CD4^+^CD25^high+^ thymocytes and splenocytes after negative selection of CD4^+^ cells followed by CD25^+^ positive selection of Treg cells. Foxp3 intracellular staining was used to test the purity of Treg subpopulations. The figure shows representative flow cytometric plots

**Fig. 5:**
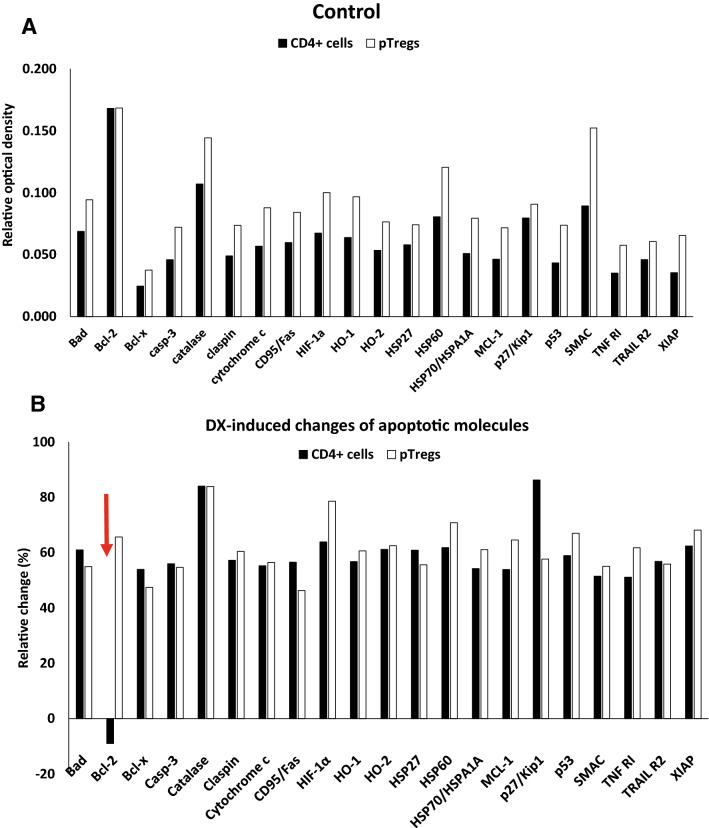
**a** Treg cells express higher level of apoptosis-related proteins than CD4^+^ T cells. Analysis of 21 apoptosis-related protein expression in the cell lysates of untreated, sorted pTreg cells and CD4^+^ splenocytes. Bars show the average OD values of each analyte from at least three independent experiments (n = 6).** b** Effect of DX treatment on the expression of 21 apoptosis-related molecules in T cell subpopulations. Protein arrays were used to detect the in vitro DX treatment (4 h) induced changes of apoptosis-related molecules in cell lysates of sorted CD4^+^ T cells and pTreg cells. Bars show the DX-induced relative changes (%) of the average OD values of each analyte compared to untreated controls from at least three independent experiments (n = 6). Relative changes were calculated with the following formula: [(OD_DX_ – OD_Ctrl_)/OD_Ctrl_] × 100%

To further investigate the different apoptosis sensitivity of CD4^+^ T cells a Tregs, we determined the pro- and anti-apoptotic molecule expression in sorted splenic CD4^+^ T cell and pTreg cells. A membrane-based antibody array was used for simultaneous detection of 21 apoptosis-related proteins in control and DX-treated samples. The purity of the sorted CD4^+^CD25^high+^ T cells was determined by flow cytometric detection of Foxp3 staining (Fig. 4). The low tTreg cell ratio and absolute cell count in the thymus did not allow us to isolate sufficient number of tTreg cells for the apoptosis protein array. When we analyzed the expression of apoptosis-related proteins in control, untreated pTreg cells, we found higher expression of all apoptosis-related proteins than that observed in CD4^+^ T cells. In untreated Tregs and CD4 + T cells, the Bcl-2 expression was similarly high, compared to other anti-apoptotic molecules like Bcl-xL (Fig. 5a).

Then we compared the effect of 4 h in vitro DX treatment on the 21 apoptosis-related molecules by analyzing the relative changes of their expression in Tregs and CD4^+^ T cells. In Tregs, we observed increased expression of all apoptosis-related molecules after 4 h DX treatment. Similar elevation could be detected in CD4^+^ T cells, except that DX treatment caused downregulation of the expression of the anti-apoptotic Bcl-2 molecule. This opposite change in the expression of Bcl-2 upon DX-treatment could explain the different apoptosis-sensitivity of the two cell populations (Fig. 5b).

### Effect of DX on the Ca^2+^ signal in CD4^+^ T cells and Treg cells

Calcium acts as a second messenger in many cell types, including lymphocytes. Resting lymphocytes maintain a low concentration of Ca^2+^. However, engagement of antigen receptors like TCR in T cells induces calcium influx from the extracellular space by several routes. Therefore, we examined how the short-term high dose in vitro DX treatment affects the intracellular free Ca^2+^ level and the TCR-induced Ca^2+^ signal in thymic and splenic CD4^+^ T cells and Treg cells.

**Fig. 6:**
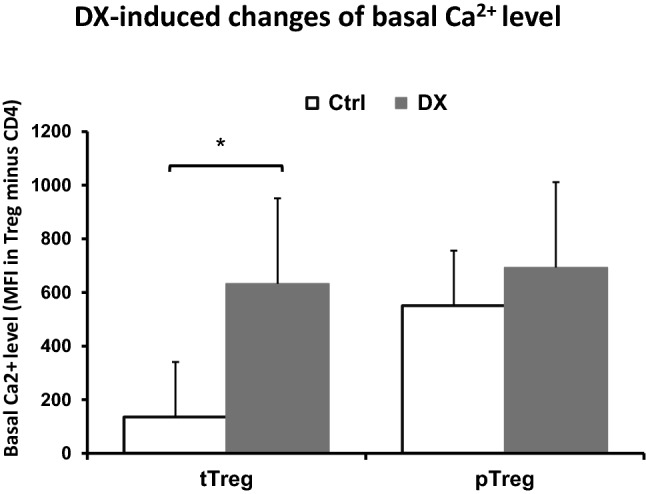
Effect of DX on basal Ca^2+^ levels in tTreg and pTreg cells. Short-term (30 min) DX treatment induced elevation of basal cytosolic free Ca^2+^ levels in thymic Treg cells. In both organs, the baseline Ca^2+^ levels were higher in Treg cells than in the corresponding CD4^+^ T cells. The columns show the Fluo 3-AM fluorescence intensities (MFI) of Tregs minus MFI of CD4^+^ T cells. Data represent mean ± SEM (n = 4). Significant change is indicated with asterisk (*p* < 0.05)

We performed cell surface labeling of thymocytes and splenocytes with anti-CD4, anti-CD8, and anti-CD25 antibodies followed by Fluo 3-AM loading for detection of cytosolic free Ca^2+^ signal changes by flow cytometry. The basal cytosolic free Ca^2+^ levels were determined in gated thymic and splenic CD4^+^ T cells and Treg cells by measuring the Fluo 3-AM fluorescence intensities (MFI). We found that in untreated samples the basal Ca^2+^ level was higher in splenic CD4^+^ T cells and pTreg cells than in CD4 SP thymocytes and tTreg cells (Fig. 6). In the control samples of both organs, Tregs showed higher tendency of basal Ca^2+^ levels than their corresponding CD4^+^ T cell populations, but this difference was not significant. Short-term (30 min) DX treatment caused significant elevation of basal cytosolic Ca^2+^ level in tTreg cells, but not in pTreg cells (Fig. 6).

**Fig. 7:**
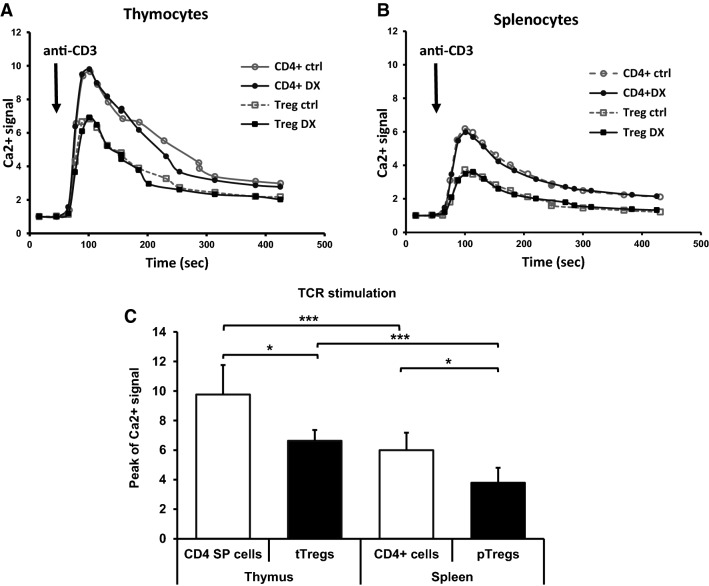
Calcium response is low in thymic and splenic Treg cells. Effect of TCR (anti-CD3) stimulation in thymic CD4 SP and tTreg cells was measured in the presence or absence of DX (**a**). Calcium influx kinetics in splenic CD4^+^ and Treg cells upon specific TCR-mediated activation (anti-CD3) in the presence or absence of DX (**b**). Data were calculated as the relative ratio of calcium values of Fluo 3-AM fluorescence intensities (median). Columns show the relative ratio of peak to baseline calcium flux measured as Fluo 3-AM fluorescence intensities (MFI) (**c**). The graphs show the mean ± SEM of at least three independent experiments (n = 9). Significant changes are indicated with asterisk (*p < 0.05, ***p < 0.001)

Next, we measured the kinetics of Ca^2+^ signal in control and DX-pretreated thymic and splenic CD4^+^ T cells and Treg cells after TCR stimulation with anti-CD3. In both organs, TCR stimulation caused lower Ca^2+^ signal in Treg cells than in CD4^+^ T cells. The Ca^2+^ signal was higher in thymic CD4 SP cells and tTreg cells than in splenic CD4^+^ T cells and pTregs, respectively. Short-term (30 min) DX pretreatment did not cause significant changes in the TCR stimulation-induced Ca^2+^ signal of the cells (Fig. 7a, b).

In order to gain further insight into TCR induced Ca^2+^ signal, we compared the peak values of Ca^2+^ signal in thymic and splenic CD4^+^ T cells and Tregs. After TCR stimulation, CD4 SP thymocytes showed significantly higher peak Ca^2+^ level than the corresponding tTreg cells, and similarly, splenic CD4^+^ T cells showed significantly higher Ca^2+^ peak value than pTreg cells. When comparing the peak Ca^2+^ values of thymic to splenic T cells, we found significantly higher Ca^2+^ levels in CD4 SP cells than in splenic CD4 + T cells, and higher peak Ca^2+^ levels in tTregs than in splenic pTregs (Fig. 7c).

**Fig. 8:**
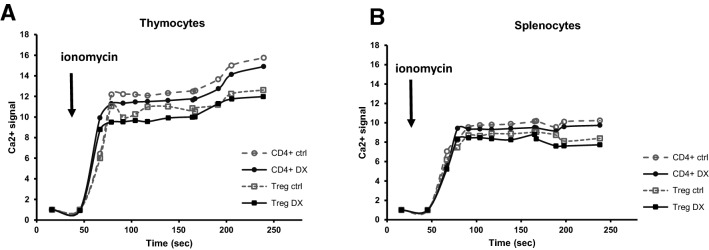
Ionomycon-induced calcium influx kinetics is lower in splenic T cells. Time-kinetics of Ca^2+^ signal was measured in thymic CD4 SP and tTreg cells (**a**) and in splenic CD4^+^ T cells and pTregs **(b**) in the presence or absence of DX. Data shown are representative (n = 9), and are presented as relative values of Fluo 3-AM fluorescence intensities (median)

We also analyzed the ionomycin-induced Ca^2+^ signal in thymic and splenic T cells and Tregs. We observed a rapid increase in the Ca^2+^ signal in all T cell subtypes examined, but the elevation was lower in splenic T cells. In both thymic and splenic Treg cells, ionomycin induced lower Ca^2+^ influx than in CD4^+^ T cells (Fig. 8a). These data are in agreement with those in the literature describing that thymocytes are more sensitive to Ca^2+^ ionophores, while memory T cells are resistant. This suggests that Ca^2+^ channels in immature thymocytes allow higher influx Ca^2+^ from the extracellular into the intracellular space. As a result of 30 min high-dose DX treatment, the nonspecific Ca^2+^ signal showed a slight, but not significant decrease both in splenic and thymic T cells, which might be due to the direct membrane stabilizing effect of DX (Fig. 8a, b).

## Discussion

Earlier we reported on the effect of in vivo administered DX on the cellular composition of lymphoid organs in mice; we found that the ratio of thymic tTregs significantly increased after DX treatment. However, this was only a relative increase, due to the selective death of GC-sensitive DP thymocytes. The absolute numbers of tTreg did not change, suggesting that these cells are resistant to the DX-induced apoptotic process [24]. We also showed previously in an in vitro study that in DP cells the ligand-bound GRs translocate into the mitochondria after 30 min, resulting in the induction of the intrinsic apoptotic pathway activation [8].

In the current study, we wanted to analyze and compare the early apoptotic processes of splenic and thymic CD4^+^ T cells and Tregs and find out which apoptosis pathway is activated as a result of GC hormone treatment. For this purpose, first we performed Annexin V labeling followed by detection of active caspases of the intrinsic and extrinsic apoptosis pathways before and after high dose in vitro (10^–6^ M) DX treatment (30 min–6 h). We observed the strongest activation of both extrinsic and intrinsic apoptosis pathways in thymic CD4 SP cells resulting in increased effector caspase-3 activation and significant elevation of the ratio of Annexin V positive cells after 4 h of treatment. In splenic CD4^+^ T cells the same trend of caspase activation could be observed, but this increase was three times lower than in thymic CD4 SP cells. This suggests that thymic CD4 SP cells are more sensitive to DX treatment than splenic CD4^+^ T cells, but less sensitive than DP thymocytes reported earlier [8]. In contrast with this, in pTregs we detected the activation of the extrinsic pathway (caspase-8 and the effector caspase-3) only after 3 h of treatment, while in tTregs minimal caspase-3 activation could be observed after 6 h of DX treatment without significant changes of Annexin V labeling. We would like to note that Annexin V binding in apoptotic cells reflects the inactivation of flippase and activation of scramblase enzymes by active caspase-3, which results in the accumulation of phosphatidylserine molecules on the external surface of the plasma membrane [27]. Our observation that caspase-3 activation was highest in DX-treated CD4 SP cells may explain that only these cells showed a significant increase of Annexin V binding. The high starting basal level and the time-dependent increase of Annexin V positivity in the control, untreated thymocytes could be a result of negative selection induced apoptosis occurring in vivo in the thymus. Additionally during the positive selection in the thymus cells are exposed to endogenous GC hormone produced by the cortical epithelial cells [28]. Thus, it can be hypothesized that the relative resistance of thymic tTregs to DX may be due to selection of these cells towards a more GC-resistant phenotype. Only a few studies have investigated the effect of GCs on the apoptotic pathways of CD4^+^ T cells and Tregs. Most of the studies are inconsistent since different doses of GCs were used, and the markers for Treg identification varied. In rodents, it has been reported that the ratio of Tregs increased in peripheral blood and secondary lymphoid organs after DX treatment [29, 30]. In asthma [31] and multiple sclerosis [32] models GCs caused a decrease of the number of Tregs. In human studies, an increase of circulating Treg cell number was reported after GC treatment of patients with asthma [33], and systemic lupus erythematosus [34, 35]. Other research groups found opposite results in similar diseases [36, 37].

We also investigated the expression of 21 pro- and anti-apoptotic molecules in CD4^+^ splenocytes and pTreg cells and found higher level of these molecules in pTreg cells. At first sight, it may seem paradoxical that the same cell population (pTreg) possesses high levels of both pro- and anti-apoptotic molecules. Published data suggest that pTreg cells are a highly dynamic and responsive cell population [38]. The Foxp3^+^ Treg cell lineage commitment is a gated event and peripheral proliferation and apoptosis are the primary determinants of Treg cell fate and population size. Several explanations might exist for the necessity of a high turnover (and high levels of molecules involved in apoptosis control) of the Treg cell pool. First the chronic proliferation caused by TCR stimulation via self-antigen stimulation may necessitate compensatory apoptosis. The pro-apoptotic effect of Foxp3 expression could lead to greater basal apoptosis levels in Treg cells, which may require compensatory proliferation during IL-2 deprivation process. The high Treg turnover may be required for sufficient regulatory function and immunometabolic plasticity [38–40]. Our current data may reflect the complex and highly regulated features of Treg cell survival and apoptosis, which is influenced by GC hormone, since in vitro DX treatment (4 h) caused opposite changes in the expression of Bcl-2 molecules in Tregs and CD4^+^ T cells. The majority of apoptosis-related molecules increased in both cell populations except the highly elevated Bcl-2 in Tregs and diminished expression in CD4^+^ T cells. The ligand-bound GR is a transcription factor that functions by regulating the expression of GC responsive genes in a positive or negative manner in different cell types of the immune system [3, 41]. Thus, it may not be surprising that GCs have distinct biological effects in the thymic and splenic T cell subpopulations, and affect the functioning Treg cells [4, 26, 42]. Our current data regarding the effects of GCs on the levels of apoptosis-related proteins suggest that genomic (and not mitochondrial) GR effects may dominate in pTregs and CD4^+^ splenocytes.

To further clarify the differences in the apoptosis sensitivity of Treg cells and conventional T cells to GC hormones, we measured the DX-induced changes of the cytosolic Ca^2+^ levels. In untreated thymic and splenic Treg cells the basal cytosolic free Ca^2+^ levels tended to be higher than in conventional CD4^+^ T cells. Short-term (30 min) DX treatment further increased the basal Ca^2+^ level in thymic tTreg cells, suggesting that GC hormone modulates the TCR-mediated response of Treg cells. During the negative selection, the affinity of TCR to antigen (MHC + self-peptide) determines the fate of the surviving CD4 SP cells. Cells with high affinity TCR die, those with medium affinity differentiate into tTreg cells, and those with low affinity TCR give rise to naïve CD4^+^ T cells. The TCR-mediated selection process in vivo can determine the high basal cytosolic Ca^2+^ level observed in the untreated thymic Treg cells and their lower TCR-induced Ca^2+^ signal.

Specific Ca^2+^ signaling pathways play an important role in apoptosis induction and/or regulation, which occurs through endoplasmic reticulum (ER) Ca^2+^ stress, and cytosolic and mitochondrial Ca^2+^ overload. Excessive cytoplasmic Ca^2+^ has been linked to apoptosis via activation of Ca^2+^/calmodulin-dependent calcineurin, which leads to dephosphorylation of Bad, that in turn promotes translocation of Bcl-2-associated X protein (Bax) to mitochondria, resulting the induction of intrinsic apoptotic signaling cascade and apoptosis [43]. DX treatment resulted in robust increase of Bcl-2 expression in Treg cells, which suggests that in Treg cells these molecules result in the inhibition of the intrinsic, mitochondrial apoptotic pathway. Treg cells are responsible for self-tolerance, and although they continuously encounter self-antigens through their TCR, they must be resistant to TCR-induced stimulation. Regarding the Ca^2+^ signal kinetics, TCR (anti-CD3) stimulation resulted in lower peak values of Ca^2+^ signal in Treg cells, compared to CD4^+^ T cells of both organs. The lowest peak of Ca^2+^ signal in pTreg cells, a mixed Treg cell population selected for suppressing the immune response against self-antigens and non-pathogenic foreign antigens in the periphery could reflect the low responsiveness of these cells to TCR stimulation. In lymphocytes, following antigen binding to the TCR, the intracellular Ca^2+^ concentration can increase to ~ 1 µM. Antigen receptor (TCR) stimulation induces activation of STIM1 and STIM2, which in turn leads to the opening of Ca^2+^ release-activated Ca^2+^ (CRAC) channels, resulting in the influx of Ca^2+^ from the extracellular into the intracellular space [44]. CRAC channelopathies can lead to immunodeficiency and autoimmunity [45], underlying the importance of the Ca^2+^ signaling in Treg cell function.

Ionomycin caused the influx of Ca^2+^ from the extracellular space, which was slightly inhibited by DX treatment. These data suggest that short-term, high dose DX treatment may exert direct membrane stabilizing effect that may influence the Ca^2+^ transporter function of ionomycin ionophore across the lipid membrane. Further, this membrane-stabilizing effect of DX may influence the function of ion-channels that are responsible for maintaining the basal intracellular Ca^2+^ levels in Treg cells. Kircher et al. reported on the calcium signaling of in vitro polarized human effector CD4^+^ T cells isolated from the peripheral blood [46]. They found that the Ca^2+^ profiles of Th1, Th2 and Th17 cells were distinct, and were shaped by the intensity of stimulation. Our data, mentioned above, also suggest that tTreg and pTreg cells show Ca^2+^ signals distinct from those in other CD4^+^ T cell subtypes, however our data are not directly comparable to those by Kircher et al. due to differences in experimental conditions (e.g. mouse thymic and splenic Treg cells versus in vitro polarized human peripheral blood Treg cells). In a recent elegant study, Vaeth et al. showed that in mice tissue- resident and follicular Treg cell differentiation is regulated by Ca^2+^ influx via Ca^2+^ release-activated Ca^2+^ (CRAC) channels [47]. They found that deletion of *Stim1* and *Stim2* genes in mature Treg cells abolished Ca^2+^ signaling and prevented their differentiation into follicular Treg and tissue-resident Treg cells. In the absence of STIM1/STIM2 in Treg cells, mice developed a broad spectrum of autoantibodies and fatal multiorgan inflammation. This study establishes a critical role of CRAC channels in controlling the lineage identity and effector functions of Treg cells [47].

To conclude, our study adds to the growing literature on the effect of GCs hormones on the cell death mechanisms and Ca^2+^ signaling of Treg cells that play crucial roles in functioning of the immune system both in health and multiple types of diseases, including autoimmunity and cancer. In Treg cells GC-induced upregulation of the Bcl-2 expression leads to the inhibition of the mitochondrial apoptotic pathway that promotes their survival. Further elevation of the originally high basal Ca^2+^ level in Treg cells may explain their relative resistance to TCR-mediated signal when compared to CD4^+^ T cells. Better understanding of the GC-induced changes in apoptosis-related proteins may be of relevance to future studies aimed at harnessing Treg cells as therapeutic targets in autoimmune diseases [48] and cancer [20].

## Abbreviations

BSA: Bovine serum albumin
Ctrl: Control
DMSO: Dimethyl sulfoxide
DP: Double positive
DX: Dexamethasone
GC: Glucocorticoid
HIF-1α: Hypoxia-inducible factor 1-alpha
HO-1: Heme oxygenase 1
HSP60: Heat shock protein 60 kDa
iTreg: Induced regulatory T cell
MCL-1: Induced myeloid leukemia cell differentiation protein Mcl-1
NaN_3_: Sodium azide
PBS: Phosphate buffered saline
pTreg: Peripheral regulatory T cell
RT: Room temperature
SMAC: Second mitochondria-derived activator of caspase
SP: Single positive
TCR: T cell receptor
Th: Helper T cell
TNFRI: Tumor necrosis factor receptor I
TRARIL R2: Tumor necrosis factor receptor superfamily member 10B
tTreg: Thymic regulatory T cell
XIAP: X-linked inhibitor of apoptosis
